# Assessing the impact of COVID-19 on HIV care cascade for people living with HIV in Ethiopia: a retrospective longitudinal study

**DOI:** 10.1136/bmjopen-2024-084244

**Published:** 2024-11-01

**Authors:** Abebe Feyissa Amhare, Mingwang Shen, Zhuoru Zou, Ruyi Xia, Jing Han, Liangmin Gao, Lei Zhang

**Affiliations:** 1 China-Australia Joint Research Center for Infectious Diseases, School of Public Health, Xi'an Jiaotong University, Health Science Center, Xi'an, Shaanxi, China; 2 College of Health Science, Salale University, Fitche, Ethiopia; 3 Xi'an Jiaotong University Health Science Center, Xi'an, Shaanxi, China; 4 Institute for International and Area Studies, Tsinghua University, Beijing, People's Republic of China; 5 School of Translational Medicine, Faculty of Medicine, Nursing and Health Sciences, Monash University, Melbourne, VIC, Australia; 6 Artificial Intelligence and Modelling in Epidemiology Program, Melbourne Sexual Health Centre, Alfred Health, Melbourne, Victoria, Australia

**Keywords:** COVID-19, HIV & AIDS, Health Services

## Abstract

**Abstract:**

**Objective:**

The study aimed to investigate the impact of COVID-19 on the cascade of HIV care for people living with HIV (PLHIV) in Ethiopia.

**Design:**

A retrospective longitudinal study.

**Setting:**

The study was conducted in North Showa Zone, Ethiopia, from pre-COVID-19 (January 2018–December 2019) and during COVID-19 (January 2020–December 2021).

**Participants:**

We identified 61901 records from 3925 PLHIV, of which 23 848 were recorded during the pandemic.

**Main outcome measure:**

We investigated indicators from four stages of the HIV care cascade, including HIV screening and diagnosis, HIV care, HIV treatment and HIV disease progression, according to a WHO framework. The indicator changes were assessed with incidence rate ratios (IRRs).

**Results:**

For HIV screening and diagnosis, the monthly number of HIV tests experienced a 46% decline from 2520 to 1361 since the pandemic (IRR 0.553; 95% CI 0.546 to 0.561). For HIV care, the monthly number of consultations was reduced by 49.6% (from 1582 to 798) since the pandemic (IRR 0.591; 95% CI 0.581 to 0.601). Similarly, the monthly number of viral load tests was reduced by 42.8% (IRR 0.614; 95% CI 0.581 to 0.650). For HIV treatment, the number of antiretroviral therapy (ART) initiations was reduced by 27.8% and the number of ART adherence by 52.5% since the pandemic. For HIV disease progression, the monthly number of PLHIV achieving viral suppression was reduced by 61.6%, while HIV-related deaths doubled during the pandemic.

**Conclusion:**

The study highlights pandemic-induced disruptions in the cascade of care for PLHIV. Targeted interventions are necessary to protect PLHIV in public health emergencies.

**Strengths and limitations of this study**

- The study analysed 4135 medical records from three public hospitals over 4 years within the cascade of HIV care and treatment framework.
- The use of a retrospective design allowed for an assessment of adherence to HIV care and treatment before and during the COVID-19 pandemic.
- Reliance on existing medical records may have introduced incomplete data into the analysis.
- Potential unmeasured confounders may have influenced the results.
- The research setting in three public hospitals may limit the generalisability of the findings to other healthcare systems or populations.

## Introduction

 The outbreak of COVID-19 affected health systems worldwide. It had the potential to cause adverse health effects on all individuals, especially those with underlying medical conditions. Chronic illnesses like diabetes, HIV, hypertension, obesity, chronic kidney disease, cardiovascular disease and cancer have experienced the highest mortality rates since the onset of the pandemic.^1^ People living with HIV (PLHIV) are particularly vulnerable during the COVID-19 pandemic.^2^ Unfortunately, they are not receiving adequate HIV services due to severe disruptions in treatment and prevention services, resulting in delayed and inadequate medical care.^3 4^ As a result, the pandemic and related restrictions have compromised the medical follow-up and psychosocial well-being of PLHIV.^5^ Those who remain on treatment are more likely to adhere to antiretroviral therapy (ART) and achieve better health outcomes, reducing the likelihood of HIV transmission to others.^6^

During the COVID-19, there has been a significant decline in HIV testing.^2 7 8^ A study conducted in the USA comparing HIV testing in 2019 and 2020 showed a sharp drop in HIV testing during the pandemic.^9^ HIV testing is a critical tool in preventing the transmission of HIV and also plays a vital role in meeting the Joint United Nations Programme on HIV/AIDS target of ending AIDS by 2030.^10^ However, reducing HIV testing during the pandemic can compromise the achievement of this target, potentially leading to increased silent transmission of HIV.^11^ The outbreak of COVID-19 has also impacted routine viral load testing and CD4 counts, which are essential indicators for healthcare providers to monitor the response of PLHIV to ART and diagnose acute HIV infections. The lack of these tests makes it difficult to determine the health status of PLHIV.^4^ Our prior qualitative research further highlighted the disruption of routine viral load testing and CD4 counts in Ethiopia caused by COVID-19.^12^

In addition to disruptions in HIV services, some PLHIV have suspended their care and ART appointments out of concern for the COVID-19 pandemic. This reduction in adherence to ART may lead to high drug resistance, which can be a significant problem.^13^ Lost follow-up appointments have been associated with an increased risk of AIDS and death in previous studies.^14 15^ ^16 17^ The discontinuation of follow-up care among PLHIV was already a widespread problem in Ethiopia even before the COVID-19 pandemic, as indicated by various studies.^1518^,^20^ However, the outbreak of COVID-19 has intensified this issue, leading to an increase in the number of PLHIV discontinuing their follow-up care in Ethiopia, as reported by recent studies.^2 12^ It is important to note that while COVID-19 may be a significant contributing factor to this issue, there are a variety of other factors that can impact HIV services, including the availability of healthcare resources, service capacity, accessibility of services and sociocultural factors.^21 22^ As such, the COVID-19 has acted as a catalyst, bringing together these various factors to accelerate and amplify the impact on the AIDS epidemic and its control in Ethiopia.^23^

The COVID-19 pandemic is anticipated to have a significant impact on low-income countries. Previous studies conducted in the Horn of Africa, including Ethiopia, have shown a decline in HIV services during the pandemic.^24^,^28^ However, while previous studies have examined the effect of COVID-19 on certain HIV indicators, the available data and samples from Ethiopia are limited. There is limited comprehensive scientific evidence on the rates of HIV service interruptions among PLHIV in Ethiopia due to the COVID-19 pandemic compared with the prepandemic period. It is crucial to identify interruption rates of HIV services among PLHIV to address the problem early. Additionally, to improve healthcare delivery and provide sustainable quality care for PLHIV during the COVID-19 pandemic and future pandemics, a better understanding of the nature and extent of HIV treatment service disruptions and the associated factors is needed.

This study aimed to investigate the impact of healthcare interruptions for PLHIV due to the COVID-19 pandemic in North Shewa, Oromia Ethiopia. Using large datasets, the study analyses the interruption rate of all HIV indicators based on the cascade of HIV care and treatment framework.^29^ This research significantly contributes to the existing literature by providing a comprehensive analysis of a wide range of HIV indicators and comparing them with the pre-COVID-19 period in Ethiopia. Unlike earlier studies that primarily focused on seasonal variations or long-term trends, our analysis offers a detailed examination of the direct impact of COVID-19 on HIV services, thereby filling a critical gap in understanding the pandemic’s effects on HIV care continuity. The results of this study will enable responsible organisations to comprehend and find solutions to decrease PLHIV healthcare disruption. It will contribute additional findings to the existing scientific literature and aid researchers in investigating further impacts of COVID-19 on HIV services. Moreover, it will provide valuable insights to policymakers for planning and allocating resources for PLHIV.

## Methods

### Study location and duration

The research was undertaken in the North Shewa Zone of the Oromia regional state, located in the northwest region of Ethiopia. The primary emphasis of the study was on three public hospitals that have been actively engaged in delivering healthcare services to PLHIV from January 2018 to December 2021. These hospitals include Salale University Comprehensive Specialised Hospital, Kuyu General Hospital and Gundo Meskel Primary Hospital. The process of data extraction took place during the period from May to July 2022.

### Study design

This study used a retrospective longitudinal descriptive study design to evaluate adherence to HIV care and treatment before (January 2018 to December 2019) and during the COVID-19 pandemic (January 2020 to December 2021). We extracted 4135 medical records of PLHIV from 1 January 2018 to December 2021 and only included complete documents or records that met HIV follow-up standards for data extraction.

### Study variables

To extract patient information from their ART cards, a questionnaire was developed based on standardised entry and follow-up forms used by ART clinics in Ethiopia. The questionnaire captured several variables, including sociodemographic characteristics and clinical conditions such as functional status, WHO stage, the number of HIV tests, HIV diagnoses, ART initiation, in-person consultations, viral load and CD4 tests, loss to follow-up (LTFU), ART adherence level, viral suppression and HIV-related deaths.

In the context of national guidelines for comprehensive HIV prevention, care, and treatment, clinical conditions and indicators are defined as follows.

*Functional statuscategorisesPLHIV into three groups*: those who can engage in daily activities, including (working) those who can independently care for themselves and use the restroom (ambulatory) and those who require assistance for even basic tasks (bedridden).

*LTFUamong PLHIV* is defined as a situation where PLHIV who was previously enrolled in care and/or receiving ART has not attended a clinical appointment or picked up their medication for a period of 3 months or more from the last expected visit date, without any formal notification of transfer, discontinuation or death.

*HIV viral load status* is assessed with a suppressed viral load indicated by a result of less than 1000 copies/mL and an unsuppressed viral load by a result of 1000 copies/mL or more.^30^

*ART adherence* is calculated as the number of pills dispensed is recorded at each visit, and the number of pills remaining is subtracted from the number dispensed to determine the number of pills taken. The adherence rate is then calculated by dividing the number of pills taken by the expected number of pills that should have been taken during the same period, usually expressed as a percentage. It can be categorised as follows.

- Good ART adherence: if PLHIV use is equal to or greater than 95% of given ART doses.
- Fair ART adherence: if PLHIV use is 85%–94% of given ART doses.
- Poor ART adherence: if PLHIV use is less than 85% of given ART doses.

*HIV-related death* is defined as a death that occurs in an individual with HIV/AIDS, where the cause of death is directly related to complications of HIV infection or its impact on the immune system. Only recorded deaths of PLHIV who died due to HIV-related complications and/or other conditions that arose because HIV had weakened their immune system were included in the study.

### Data collection

Medical history documents were collected and reviewed from selected hospitals based on the developed questionnaire. The information retrieved encompassed both pre-COVID-19 and during the COVID-19 period. Five experienced ART nurses who were trained in comprehensive HIV care and involved in the follow-up of PLHIV were selected for data collection and supervised by the principal investigator. The collected data underwent daily rigorous examination to ensure clarity and consistency. Trained data clerks then entered the information into Excel and performed data cleaning procedures before analysis.

### Cascade of HIV care and treatment

**Figure 1:**
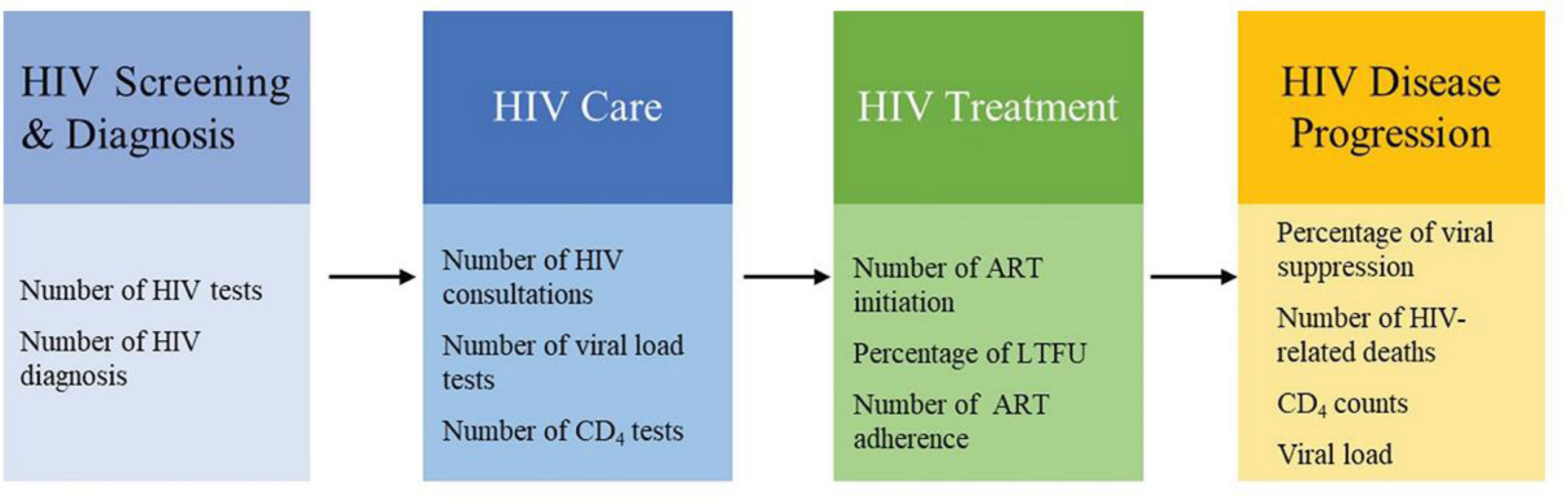
HIV care continuums. The HIV care continuum diagram has been adapted and modified according to the WHO guidelines. This modified representation highlights the stages involved in the continuum of HIV care as recommended by WHO, emphasising the critical steps from HIV diagnosis to viral suppression.^29^

The current investigation delved into the repercussions of the COVID-19 pandemic on the uninterrupted provision of HIV services, with a specific focus on the cascade of the HIV care continuum. We adapted the cascade framework and modified it following the guidelines set forth by the WHO (figure 1).^29^ The research encompassed several distinct areas of scrutiny. First, we assessed the impact of COVID-19 on HIV screening and diagnosis, conducting a comparative analysis of these measures during the COVID-19 era against the backdrop of the pre-COVID-19 period. Second, our investigation delved into the realm of HIV care, scrutinising the influence of COVID-19 on key determinants such as regular medical visits and the performance of routine laboratory tests. Third, we explored the domain of HIV treatment, evaluating the repercussions of COVID-19 on various facets of treatment, including ART initiation, continuity of care and adherence to ART. Lastly, our study investigated the impact of COVID-19 on HIV disease progression, specifically analysing key indicators such as CD4 counts, viral load levels, rates of viral suppression and HIV-related mortality.

### Data quality control

Since the data was extracted from records, the data collectors were adequately trained to extract recorded data of all PLHIV during the study period and check the integrity or completeness of the data. After collection, the data was cross-checked for consistency and completeness to ensure their quality and avoid any bias. Based on this, the recorded data of 210 PLHIV was discarded due to incomplete records.

### Data analysis

In this scientific investigation, a comprehensive approach was taken to analyse the data. First, meticulous data cleaning and entry procedures were conducted using Microsoft Excel, after which the data was exported to Stata V.17 for the subsequent statistical analysis. To provide a clear overview of the findings, descriptive statistics were conducted to present results in both numerical and percentage-based formats. Plots were used to illustrate visual trends in HIV services during COVID-19 compared with prepandemic times.

For count-based outcomes, Poisson regression models were employed to determine incidence rate ratios (IRRs) and establish their corresponding 95% CIs. The IRRs were then contrasted between the COVID-19 and pre-COVID-19 periods. To further investigate differences in CD4 counts and viral load between these two timeframes, a Mann-Whitney U test was executed. Furthermore, a logistic regression analysis was employed to explore the specific sociodemographic and clinical conditions of PLHIV that were associated with disruptions in HIV services during the COVID-19 pandemic. A p-value less than 0.05 indicated a significant outcome.

### Patient and public involvement

None.

## Results

### Sociodemographic and clinical conditions of PLHIV

This investigation examined the records of 4135 PLHIV from January 2018 to December 2021. Among these, the records of 210 PLHIV were excluded due to incomplete information, resulting in 3925 PLHIV records for analysis. Of the included PLHIV, 2435 (62%) were female, and the average age of all PLHIV was 37 years (SD±13). Most of the participants were classified as WHO stage 1 based on their clinical status (online supplemental table S1). The analysis included 61 901 records of PLHIV. Furthermore, throughout the study period, 99 296 individuals were screened for HIV. Among these individuals, 63 773 HIV tests were performed before COVID-19.

### HIV screening and diagnosis

**Figure 2:**
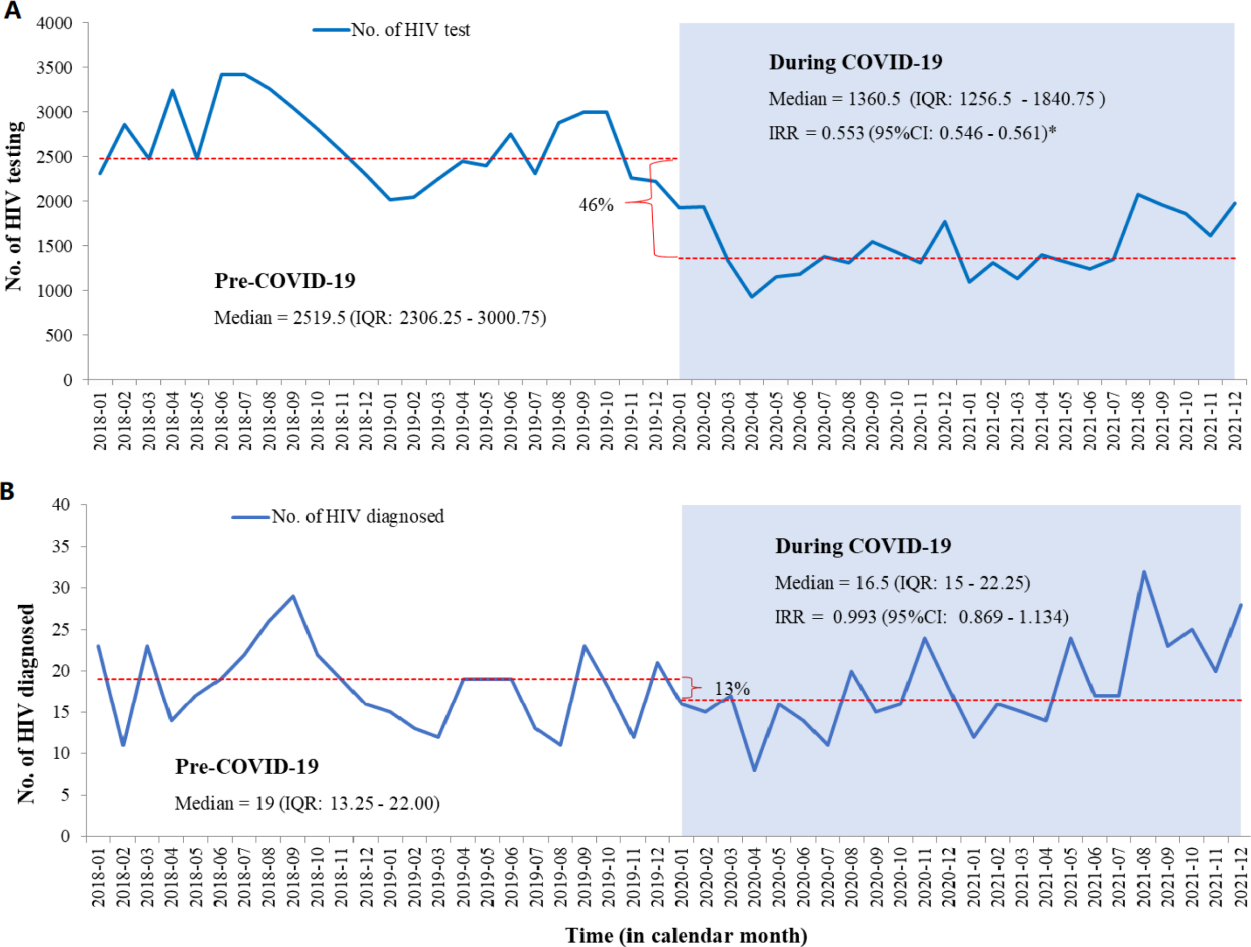
Interruption of HIV screening and diagnosis in North Showa Zone, Oromia region, Ethiopia, from 2018 to 2021. IRR, incidence rate ratio; No., number; *p<0.05.

The median of monthly HIV tests conducted was the highest in 2018 with 2832 tests (2479–3255) and in 2019 with 2353 tests (2233–2849). This reduced significantly to 1352 tests (1213–1714) in 2020 and 1375 tests (1258–1935) in 2021. Overall, the median of monthly HIV testing before COVID-19 was 2520 tests (2306–3001), which was significantly reduced by 46% to 1361 tests (1257–1841) during the COVID-19 pandemic (IRR 0.553; 95% CI 0.546 to 0.561, figure 2A).

This study also explored the impact of COVID-19 on the number of monthly HIV diagnoses. We observed a slight reduction in the number of HIV diagnoses during the pandemic. The median monthly HIV diagnosis was 19 (13.25–22.00) before the pandemic, which decreased to 17 (15–22) during the pandemic. Although this reduction corresponds to a 13% decline, it was not statistically significant (IRR 0.993; 95% CI 0.869 to 1.134, figure 2B).

### HIV care

The study found that essential HIV care components such as in-person consultations and vital tests like CD4 counts and viral load monitoring were interrupted during the COVID-19 pandemic.

**Figure 3:**
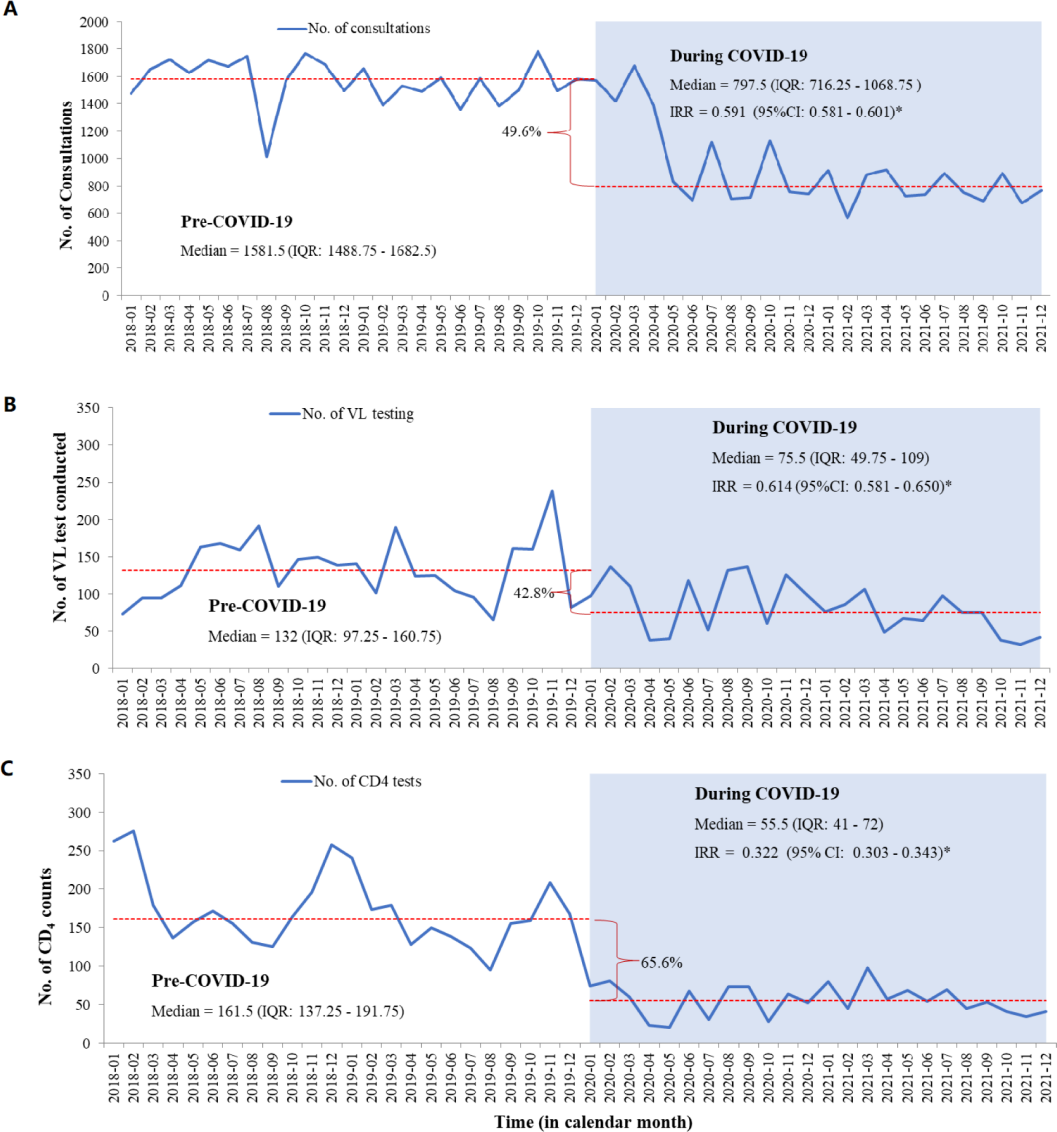
Interruption of HIV care of people living with HIV in North Showa Zone, Oromia region, Ethiopia, from 2018 to 2021. IRR, incidence rate ratio; No., number; *p<0.05.

In-person clinical consultations significantly decreased during COVID-19 compared with before. The median monthly in-person consultations dropped from 1582 (1489–1683) before the pandemic to 798 (716–1069) during the pandemic, marking a substantial 49.6% reduction (IRR 0.591; 95% CI 0.581 to 0.601, figure 3A).

We further explored whether similar declines occurred when considering participants’ sociodemographic characteristics and clinical conditions. Notably, individuals aged 25–64 years (OR 0.841; 95% CI 0.761 to 0.929) and those over 64 years (OR 0.792; 95% CI 0.676 to 0.928) were less inclined to seek medical centre visits compared with PLHIV aged under 15 years. Additionally, PLHIV in WHO clinical stage 2 were less likely to visit medical centres during the COVID-19 pandemic (OR 0.930; 95% CI 0.887 to 0.976) compared with those in WHO clinical stage 1 (online supplemental table S2).

Furthermore, the study identified that viral load testing was interrupted during the COVID-19 pandemic in the study area. Before the pandemic, the median monthly viral load tests stood at 132 (97–161), experiencing a significant reduction of 42.8% to 76 (50–109) during COVID-19 (IRR 0.614; 95% CI 0.581 to 0.650, figure 3B). Moreover, when we examined this decline while considering the sociodemographic and clinical characteristics of PLHIV who underwent viral load testing, it was observed that individuals in the age group of 15–24 years were more likely to maintain regular viral load testing during the COVID-19 period (OR 1.129; 95% CI 0.787 to 1.620) compared with those aged under 15 years (online supplemental table S2).

In addition, the pandemic significantly disrupted the provision of CD4 testing for PLHIV, resulting in a remarkable drop of over 65% compared with prepandemic testing levels. Before COVID-19, the median monthly CD4 tests stood at 162 (137–192), but during the pandemic, it plummeted to 56 (41–72) tests per month. This reduction was statistically significant (IRR 0.322; 95% CI 0.303 to 0.343, figure 3C).

When considering sociodemographic characteristics and clinical conditions, CD4 testing decreased during COVID-19 compared with prepandemic levels across sex, various age groups, functional statuses and WHO stages. Male individuals were more likely to undergo CD4 testing during COVID-19 (OR 1.175; 95% CI 1.032 to 1.337) than female individuals (online supplemental table S2).

### HIV treatment

**Figure 4:**
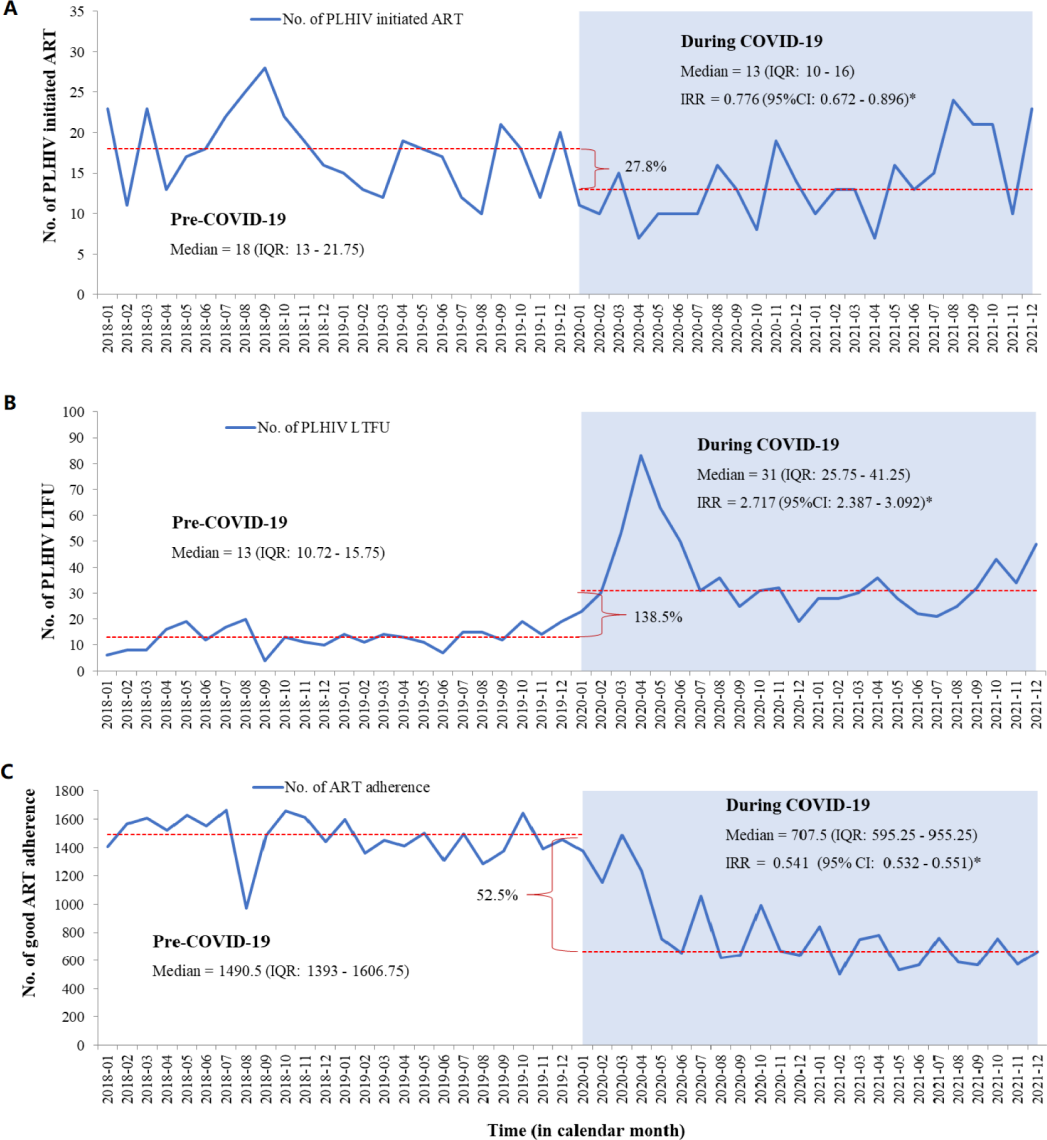
Interruption of HIV treatment of people living with HIV in North Showa Zone, Oromia region, Ethiopia, from 2018 to 2021. ART, antiretroviral therapy; IRR, incidence rate ratio; No., number; *p<0.05.

This study also explored the impact of the pandemic on HIV treatment. Before COVID-19, the median monthly number of ART initiations among individuals diagnosed with HIV was 18 (13–22), but this declined to 13 (10–16) during the pandemic. This change represented a significant 27.8% decrease in the monthly number of ART initiations (IRR 0.776; 95% CI 0.672 to 0.896, figure 4A). Furthermore, there was a significant decrease in the number of ART initiations among females living with HIV during the COVID-19 pandemic (IRR 0.740; 95% CI 0.613 to 0.893) (online supplemental table S3).

Furthermore, compared with the period preceding the COVID-19 outbreak, there was a substantial increase in individual LTFU during the pandemic. Before COVID-19, the monthly median of LTFU was 13 (11–16), but it rose to 31 (26–41) during the pandemic. The incidence rate of LTFU during the pandemic was 2.717 times higher (IRR 2.717; 95% CI 2.387 to 3.092) than before the COVID-19 outbreak (figure 4B).

The COVID-19 pandemic significantly impacted the number of PLHIV with good ART adherence. Before the pandemic, the median number of PLHIV with good ART adherence was 1491 (1393–1607). During COVID-19, this number dropped to 708 (595–955), representing a 52.5% reduction, which was statistically significant (IRR 0.541; 95% CI 0.532 to 0.551, figure 4C).

### HIV disease progression

**Figure 5:**
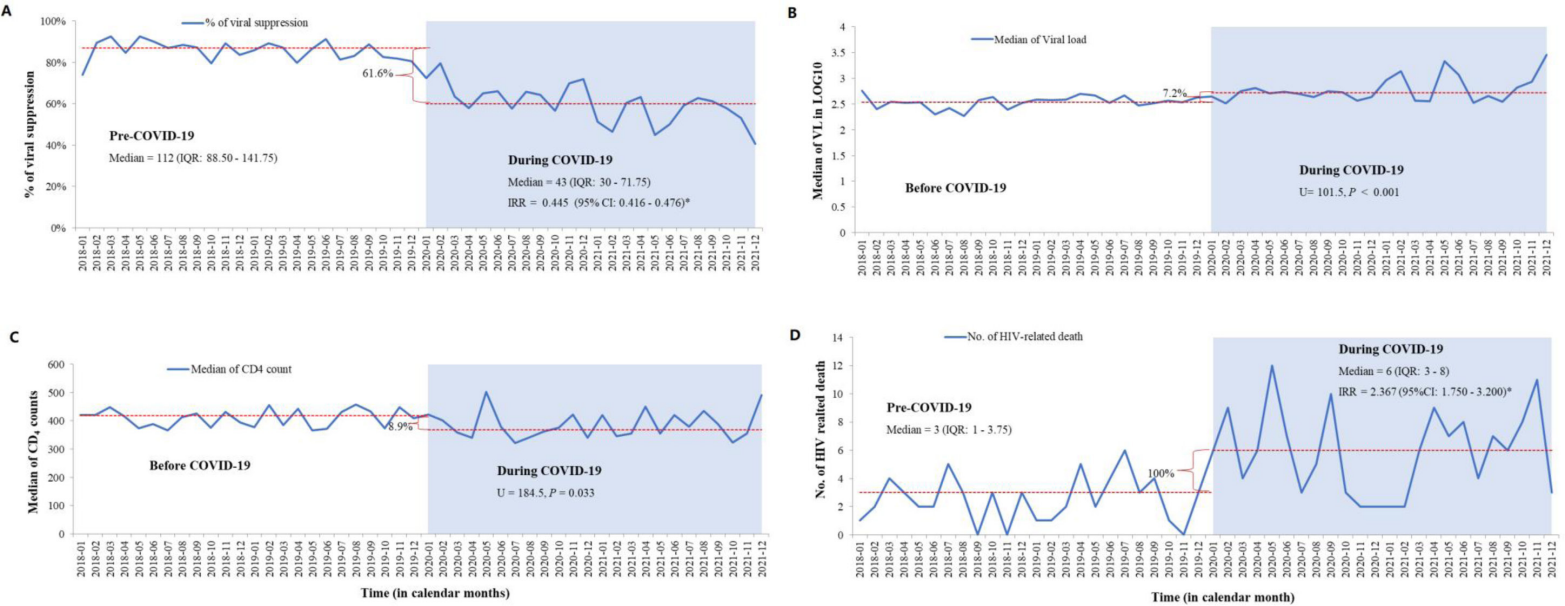
Interruption of HIV disease progression of people living with HIV in North Showa Zone, Oromia region, Ethiopia, from 2018 to 2021. IRR, incidence rate ratio; No., number; U, Mann-Whitney U test; *p<0.05.

Moreover, this study explored the impact of COVID-19 on various aspects of HIV disease progression. Before COVID-19, the median monthly count of PLHIV with viral suppression was 112 (89–142). However, during the pandemic, this number dropped to 43 (IQR: 30–72), representing a significant 61.6% reduction in individuals with suppressed viral loads (IRR 0.445; 95% CI 0.416 to 0.476, figure 5A). This decline was consistent across sex, various age groups, functional status and different WHO stages (online supplemental table S4).

In addition, PLHIV who underwent testing displayed a considerably elevated viral load (U=101.5, p=0.001, figures5B  6A) during the pandemic. The median viral load count before COVID-19 was 352 (75–717) copies/mL and increased to 483 (73–4737) copies/mL during the pandemic. Additionally, our study explored the impact of the outbreak on CD4 counts, revealing a relative decrease in those tested (U=184.5, p=0.033, figures5C  6B). The median CD4 count before COVID-19 stood at 419 (254–584) cells/mm³, which declined to 366 (227–578) cells/mm³ during COVID-19.

**Figure 6:**
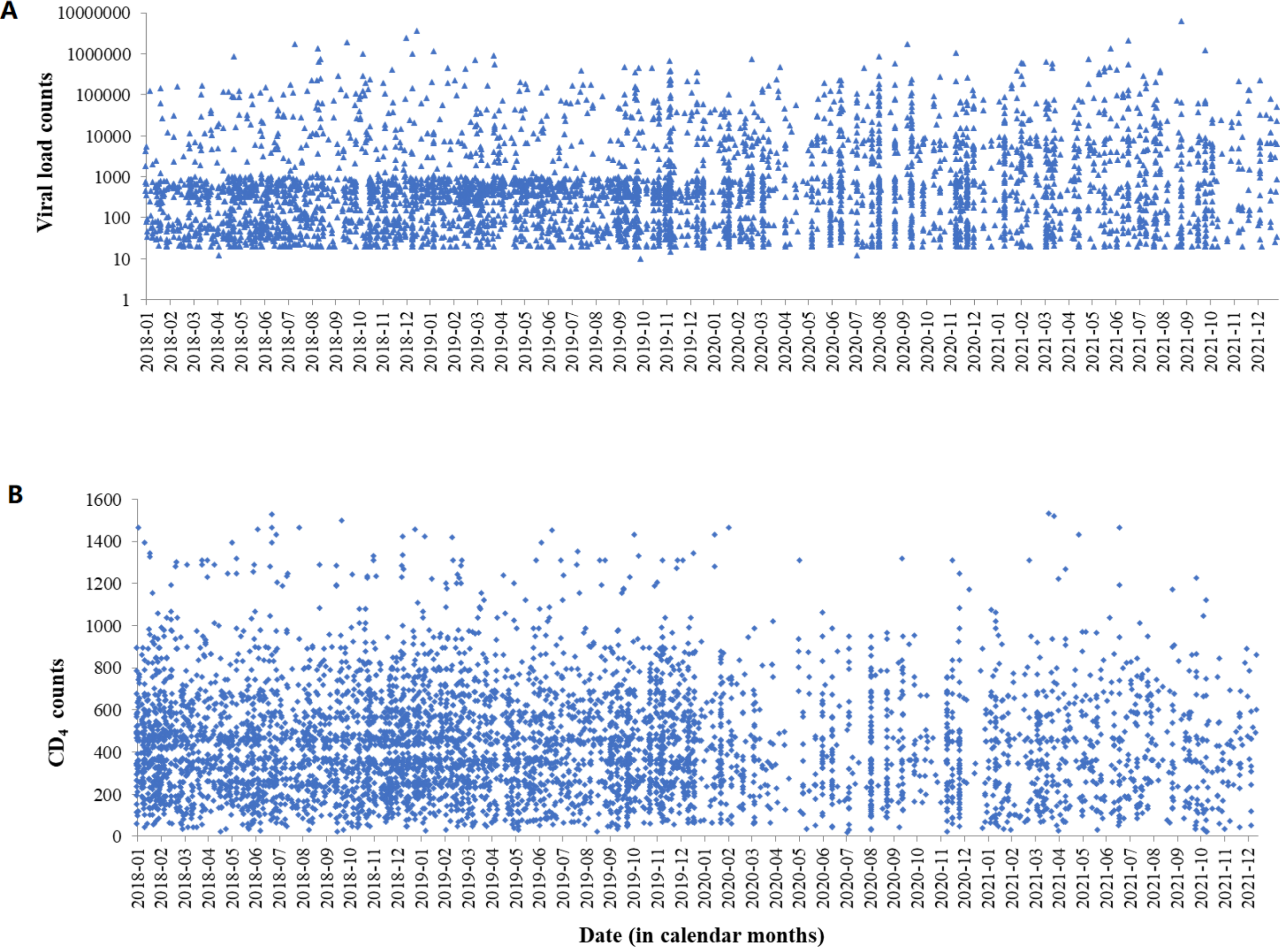
Scatter plots of viral load and CD4 counts of people living with HIV from January 2018 to December 2021 in North Showa Zone, Oromia region, Ethiopia.

Furthermore, compared with the pre-COVID-19 period, the pandemic led to a notable increase in HIV-related deaths, with a median monthly count rising from 3 (1–4) to 6 (3–8). This increase was statistically significant (IRR 2.367; 95% CI 1.750 to 3.200, figure 5D).

## Discussion

This study evaluated the impact of COVID-19 on HIV services compared with the pre-COVID-19 period, revealing significant disruptions across the HIV care continuum. HIV testing, consultation sessions and routine tests experienced substantial declines, leading to reduced ART initiation, healthcare continuity, and the number of PLHIV achieving viral suppression and adherence. As a result, there was a noteworthy increase in HIV-related mortality during the pandemic.

In Ethiopia, the COVID-19 pandemic disrupted HIV screening efforts, resulting in a decrease in the number of HIV tests conducted. Although there was a noticeable decline in HIV diagnoses, this change did not reach statistical significance. This hints at a potential shift in HIV screening strategies, possibly with less emphasis on low-risk populations. During the COVID-19 pandemic in Ethiopia, HIV testing services were limited to specific targeted groups and high-risk populations. These included sex workers, men who have sex with men, truck drivers and mobile workers, prisoners, discordant couples, pregnant women, patients with sexually transmitted infections and patients with TB. Testing was conducted exclusively at health institutions, as community outreach testing services were halted during this period.^12^ The trend of reduced HIV testing during the pandemic is consistent with findings from previous studies.^2 7 31 32^ Early HIV diagnosis allows PLHIV to start ART promptly, which is highly beneficial for those with HIV.^33 34^ Also, it can reduce HIV transmission, and lower risks of HIV-related illnesses, mortality and associated health issues.^34 35^

COVID-19 has disrupted regular HIV care and follow-up significantly, impacting key aspects such as in-person consultations, viral load testing and CD4 counts. During the pandemic, face-to-face consultations with PLHIV decreased by more than half, indicating a notable decline in consistent care provision, a trend consistent with previous studies.^8 12^ It was due to public health measures like lockdowns and social distancing, the increased risk of COVID-19 exposure for immunocompromised individuals and the strain on healthcare systems. The decrease in consultations raises concerns about poor retention in care, especially in regions without telemedicine alternatives for PLHIV.^12^ The other part of the reason for the reduction in in-person consultations among PLHIV could be the increase in patient-centred differentiated service delivery models, such as multimonth dispensing (MMD). These models aim to reduce the burden on healthcare facilities and patients by allowing for fewer visits and extended intervals between medication pickups. Ethiopia has adopted differentiated service delivery models, including MMD.^12 36^ This approach has been implemented to improve adherence to ART, reduce the frequency of clinic visits and minimise potential exposure to COVID-19.

Furthermore, viral load tests and CD4 counts among PLHIV have seen a substantial decrease since the pandemic began. Previous studies have noted routine testing disruptions and similar trends have been observed.^12 37^ These tests are vital for evaluating PLHIV’s health status and indirectly measuring their retention in HIV care.^38^ However, challenges such as laboratory staff shifting focus to COVID-19 testing and material shortages have led to reduced testing for PLHIV. This testing reduction has resulted in elevated rates of unsuppressed viral loads, linked to reduced survival among PLHIV and increased risk of HIV transmission.^39^

COVID-19 has disrupted the distribution of ART among PLHIV and compromised the quality of ART treatment. During the pandemic, there was a significant decrease in the initiation of ART compared with the prepandemic period. This trend is consistent with findings from earlier studies.^7 8^ Delayed initiation of ART has the potential to worsen treatment outcomes and increase the risk of HIV transmission.^40^ Additionally, the study revealed an elevated risk of poor ART adherence during the pandemic, which significantly impacts treatment quality. ART adherence among PLHIV during the COVID-19 pandemic may have decreased due to psychological stress and anxiety, which can disrupt daily routines, impair memory and reduce motivation, leading to forgetfulness in taking medication. These mental health challenges, combined with misinformation, changes in healthcare services, economic hardships and social isolation, further contributed to reduced adherence.^26 27 41 42^ Poor adherence to ART can trigger drug resistance,^13^ jeopardise treatment efficacy,^43^ increase the likelihood of comorbidities, exacerbate pre-existing conditions, raise healthcare costs^44^ and ultimately contribute to higher HIV/AIDS mortality rates.

Furthermore, the study found an increase in the number of PLHIV who LTFU of HIV care during COVID-19. The likelihood of being LTFU escalated considerably during the pandemic compared with the probability before the pandemic. Comparable studies have affirmed the amplified rate of LTFU HIV care during the pandemic.^3 5 45 46^ LTFU during the pandemic was increasing due to a lack of HIV services and fear of the spread of COVID-19.^47^ Another possible reason for the increased LTFU among PLHIV could be informal transfers to other health institutions, which may have also occurred before the COVID-19 pandemic. The assessment of the LTFU rate assumes significant importance in implementing measures to reinstate the standard follow-up status of PLHIV.^48^ LTFU from ART is the major contributor to attrition and further poor quality of life and death.^15 49^

COVID-19 has been linked to an escalation in HIV disease progression, marked by a decrease in viral suppression rates, a decline in CD4 counts and an increase in HIV-related mortality during the pandemic. Our analysis specifically examines viral load suppression rates among PLHIV who underwent viral load testing, ensuring that our findings accurately reflect treatment effectiveness within this group, regardless of the overall decline in facility attendance during COVID-19. While a study has reported an increase in viral load suppression rates during the pandemic,^26^ these trends may differ depending on local healthcare contexts and the characteristics of individuals who continued to access services. Our analysis aligns with the methodological framework and accurately reflects the observed data. Notably, the study revealed a significant rise in mortality among PLHIV compared with the pre-COVID-19 era. This increase in mortality could be attributed to factors such as heightened LTFU, unsuppressed viral load, and suboptimal adherence to ART, all of which are known contributors to poor health outcomes.^50 51^

However, the present study has certain limitations. First, its retrospective nature relies on existing medical records, which may contain inaccuracies or incomplete information, potentially affecting the study’s findings. Second, the research was conducted in three public hospitals in the North Shewa Zone of the Oromia regional state, which may limit the generalisability of the results to other regions or countries with different healthcare systems and demographics. Additionally, despite rigorous data quality control, 210 records were discarded due to incompleteness, potentially introducing bias or limiting the comprehensiveness of the study outcomes. However, since we used big data, the bias may not be significant. Furthermore, the analysis may be influenced by unmeasured confounders or variables affecting adherence to HIV care and treatment that were not accounted for. Lastly, the study primarily used quantitative data, which may overlook qualitative aspects and individual experiences of PLHIV, potentially missing additional insights into the challenges faced during the COVID-19 pandemic. Consequently, it is suggested that further research be carried out to investigate the combined influence of the COVID-19 pandemic and various social factors on HIV services and prevention methods.

## Conclusion

In general, this study underscores the significant disruptions in HIV services resulting from the COVID-19 pandemic. The substantial decline in HIV testing and in-person consultations during this period has had far-reaching consequences. These disruptions have led to a heightened risk of LTFU, poor adherence to ART increased unsuppressed viral load cases and elevated mortality rates among PLHIV. The implications of these interruptions in HIV care not only threaten the survival of PLHIV but also exacerbate the potential for HIV transmission and contribute to a growing disease burden in the country. These findings highlight critical gaps in the delivery of HIV care during public health emergencies, such as the one caused by COVID-19 in Ethiopia. It is imperative for the government to proactively prepare for future health crises and prioritise the specific needs of PLHIV in emergency response plans.

## supplementary material

10.1136/bmjopen-2024-084244 online supplemental table 1

## Data Availability

Data are available upon reasonable request.
