# Supplementary material for: Assessing the impact of COVID-19 on HIV care cascade for people living with HIV in Ethiopia: a retrospective longitudinal study
Source: BMJ Open. 2024 Nov 1;14(10):e084244. doi: 10.1136/bmjopen-2024-084244 (PMC11529552; doi:10.1136/bmjopen-2024-084244)
Supplement: online supplemental table 1 [file bmjopen-14-10-s001.pdf]

# Supplemental material

# Appendix

**Table S1.** Socio-demographic and Clinical Conditions of PLHIV

| **Baseline Variables** | | **Frequency n (%)** |
|---|---|---|
| Sex | Female | 2435 (62.0) |
| | Male | 1490 (38.0) |
| Age | < 15 | 240 (6.1) |
| | 15-24 | 305 (7.8) |
| | 25-64 | 3305 (84.2) |
| | > 64 | 75 (1.9) |
| Functional status | Working | 3500 (90.4) |
| | Ambulatory | 320 (8.3) |
| | Bedridden | 51 (1.3) |
| Development milestone | Appropriate | 41 (75.9) |
| | Delay | 10 (18.5) |
| | Regression | 3 (5.6) |
| WHO stage | One | 2825 (72.0) |
| | Two | 549(14.0) |
| | Three | 461 (11.7) |
| | Four | 90 (2.3) |

**Table S2.** Interruption of HIV care during COVID-19 Pandemic based on Socio-demographic Characteristics and Clinical Conditions of PLHIV

| **Indicators** | **Baseline Variables** | | **Before COVID-19 (Jan 2018 to Dec 2019)** | **During COVID-19 (Jan 2020 to Dec 2021)** | **Change in percentage (%)** | **IRR (95%CI), Comparing indicators before and during COVID-19** | **OR (95%CI), Comparing subgroups with the reference group** |
|---|---|---|---|---|---|---|---|
| **Number of in-person consultations** | Sex | Female | 24005 | 14504 | -39.6 | 0.604 (0.592 - 0.617)*** | ref |
| | | Male | 13563 | 8234 | -39.3 | 0.607 (0.591 - 0.624)*** | 1.001 (0.967 - 1.037) |
| | Age | < 15 | 2869 | 1852 | -35.4 | 0.646 (0.609 - 0.684)*** | ref |
| | | 15-24 | 2634 | 1684 | -36.1 | 0.639 (0.601 - 0.679)*** | 0.897 (0.800 - 1.007) |
| | | 25-64 | 31365 | 18806 | -40.0 | 0.599 (0.589 - 0.611)*** | 0.841 (0.761 - 0.929)*** |
| | | > 64 | 700 | 396 | -43.4 | 0.566 (0.500 - 0.639)*** | 0.792 (0.676 - 0.928)** |
| | Functional status | Working | 33448 | 20263 | -39.4 | 0.606 (0.595 - 0.616)*** | ref |
| | | Ambulatory | 3599 | 2113 | -41.3 | 0.587 (0.556 - 0.619)*** | 0.874 (0.800 - 0.954)** |
| | | Bedridden | 82 | 46 | -43.9 | 0.561 (0.391 - 0.805)** | 1.030 (0.694 - 1.529) |
| | Development | Appropriate | 328 | 227 | -30.8 | 0.692 (0.584 - 0.819)*** | ref |

| | | | | | | | |
|---|---|---|---|---|---|---|---|
| | milestone | Delay | 79 | 67 | -15.2 | 0.848 (0.612 - 1.174) | 1.109 (0.759 - 1.619) |
| | | Regression | 32 | 22 | -31.3 | 0.688 (0.399 - 1.183) | 0.862 (0.484 - 1.536) |
| | WHO stage | One | 26901 | 16509 | -38.6 | 0.614 (0.602 - 0.626)*** | ref |
| | | Two | 5618 | 3182 | -43.4 | 0.566 (0.542 - 0.592)*** | 0.930 (0.887 - 0.976)** |
| | | Three | 4589 | 2798 | -39.0 | 0.609 (0.582 - 0.639)*** | 1.016 (0.963 -1.072) |
| | | Four | 460 | 249 | -45.9 | 0.541 (0.464 - 0.631)** | 0.885 (0.745 - 1.052) |
| **Number of viral load test** | Sex | Female | 2020 | 1192 | -40.99 | 0.590 (0.549 - 0.634)*** | ref |
| | | Male | 1164 | 764 | -34.36 | 0.656 (0.599 - 0.719)*** | 1.108 (0.985 - 1.246) |
| | Age | < 15 | 209 | 129 | -38.28 | 0.617 (0.495 - 0.769)*** | ref |
| | | 15-24 | 195 | 136 | -30.26 | 0.697 (0.560 - 0.868)** | 1.129 (0.787 - 1.620) |
| | | 25-64 | 2716 | 1660 | -38.88 | 0.611 (0.574 - 0.649)*** | 0.995 (0.740 - 1.337) |
| | | > 64 | 64 | 31 | -51.56 | 0.484 (0.315 - 0.744)** | 0.764 (0.455 - 1.284) |
| | Functional | Working | 2906 | 1790 | -38.40 | 0.616 (0.581 - 0.653)*** | ref |

| | | | | | | | |
|---|---|---|---|---|---|---|---|
| | status | Ambulatory | 237 | 143 | -39.66 | 0.603 (0.490 - 0.743)*** | 0.922 (0.701 - 1.212) |
| | | Bedridden | 12 | 3 | -75.00 | 0.25 (0.071 - 0.886)* | 0.463 (0.117 - 1.827) |
| | Development milestone | Appropriate | 21 | 14 | -33.33 | 0.667 (0.339 - 1.311) | ref |
| | | Delay | 6 | 5 | -16.67 | 0.833 (0.254 - 2.731) | 1.309 (0.305 - 5.611) |
| | | Regression | 2 | 1 | -50.00 | 0.5 (0.045 - 5.514) | 1.021 (0.075 - 13.833) |
| | WHO stage | One | 2264 | 1368 | -39.58 | 0 .604 (0.565 - 0.646)*** | ref |
| | | Two | 478 | 304 | -36.40 | 0.636 (0.551 - 0.734)*** | 1.051 (0.895 - 1.235) |
| | | Three | 397 | 263 | -33.75 | 0.662 (0.567 - 0.774)*** | 1.116 (0.933 - 1.335) |
| | | Four | 45 | 21 | -53.33 | 0.467 (0.278 - 0.783)** | 0.902 (0.506 - 1.608) |
| **Number of $CD_4$ test** | Sex | Female | 2682 | 819 | -69.5 | 0.305 (0.282 - 0.330)*** | ref |
| | | Male | 1447 | 512 | -64.6 | 0.354 (0.319 - 0.391)*** | 1.175 (1.032 - 1.337)* |
| | Age | < 15 | 324 | 83 | -74.4 | 0.256 (0.201 - 0.326)*** | ref |
| | | 15-24 | 266 | 104 | -60.9 | 0.391 (0.312 - 0.490)*** | 1.325 (0.879 - 1.998) |

| | | | | | | |
|---|---|---|---|---|---|---|
| | 25-64 | 3473 | 1128 | -67.5 | 0.325 (0.303 - 0.347)*** | 1.126 (0.795 - 1.594) |
| | > 64 | 66 | 16 | -75.8 | 0.242 (0.140 - 0.419)*** | 0.794 (0.415 - 1.517) |
| Functional status | Working | 3699 | 1209 | -67.3 | 0.327 (0.306 - 0.349)*** | ref |
| | Ambulatory | 364 | 103 | -71.7 | 0.283 (0.227 - 0.352)*** | 0.872 (0.641 - 1.185) |
| | Bedridden | 11 | 9 | -18.2 | 0.818 (0.339 - 1.974) | 2.427 (0.842 - 6.998) |
| Development milestone | Appropriate | 38 | 5 | -86.8 | 0.132 (0.052 - 0.334)*** | ref |
| | Delay | 9 | 4 | -55.6 | 0.444 (0.137 - 1.443) | - |
| | Regression | 3 | 0 | - | - | - |
| WHO stage | One | 2929 | 913 | -68.8 | 0.312 (0.289 - 0.336)*** | ref |
| | Two | 639 | 214 | -66.5 | 0.335 (0.287 - 0.391)*** | 1.084 (0.912 - 1.289) |
| | Three | 511 | 181 | -64.6 | 0.354 (0.298 - 0.419)*** | 1.170 (0.965 - 1.417) |
| | Four | 50 | 23 | -54.0 | 0.46 (0.281 - 0.754)** | 1.073 (0.577 - 1.997) |

IRR, Incidence Risk Ratio; OR, Odds ratio; **P*<0.05; ***P*<0.01; ****P*<0.001; ref, reference; Negative sign (-) indicate percentage reduction

**Table S3.** Interruption of HIV treatment during COVID-19 based on Socio-demographic Characteristics and Clinical Conditions of PLHIV

| Indicators | Baseline Variables | | Before COVID-19 (Jan 2018 to Dec 2019) | During COVID-19 (Jan 2020 to Dec 2021) | Change in percentage (%) | IRR (95%CI), Comparing indicators before and during COVID-19 | OR (95%CI), Comparing subgroups with the reference group |
|---|---|---|---|---|---|---|---|
| Number of ART Initiation | Sex | Female | 254 | 188 | -26.0% | 0.740 (0.613 - 0.893)** | ref |
| | | Male | 169 | 136 | -19.5% | 0.805 (0.642 - 1.008) | 1.134 (0.834 - 1.543) |
| | Age | < 15 | 34 | 16 | -52.9% | 0.471 (0.259 - 0.853)* | ref |
| | | 15-24 | 54 | 37 | -31.5% | 0.685 (0.451 - 1.041) | 2.415 (0.653 - 8.928) |
| | | 25-64 | 324 | 268 | -17.3% | 0.827 (0.704 - 0.972)* | 2.913 (0.841 - 10.083) |
| | | > 64 | 11 | 3 | -72.7% | 0.273 (0.076 - 0.978)* | 0.887 (0.148 - 5.329) |
| | Functional status | Working | 361 | 280 | -22.4% | 0.776 (0.664 - 0.907)*** | ref |
| | | Ambulatory | 32 | 17 | -46.9% | 0.531 (0.295 - 0.957)* | 1.321 (0.541 - 3.225) |
| | | Bedridden | 13 | 17 | 30.8% | 1.308 (0.635 - 2.692) | 3.614 (0.752 - 17.380) |
| | Development | Appropriate | 14 | 5 | -64.3% | 0.357 (0.129 - 0.992)* | ref |

| | | | | | | | |
|---|---|---|---|---|---|---|---|
| | milestone | Delay | 3 | 4 | 33.3% | 1.333 (0.298 - 5.957) | 2.038 (0.303 - 13.722) |
| | | Regression | 0 | 1 | - | - | - |
| | WHO stage | One | 318 | 247 | -22.3% | 0.777 (0.657 - 0.917)** | ref |
| | | Two | 35 | 34 | -2.9% | 0.971 (0.606 - 1.557) | 1.332 (0.792 - 2.241) |
| | | Three | 50 | 22 | -56.0% | 0.44 (0.266 - 0.726)** | 0.562 (0.309 - 1.022) |
| | | Four | 20 | 21 | 5.0% | 1.05 (0.569 - 1.937) | 0.456 (0.107 - 1.935) |
| **Number of LTFU** | Sex | Female | 179 | 496 | 177.1 | 2.771 ( 2.336 - 3.287)*** | ref |
| | | Male | 129 | 356 | 176.0 | 2.760 (2.256 - 3.375)*** | 1.074 (0.820 - 1.407) |
| | Age | < 15 | 17 | 54 | 217.6 | 3.176 (1.842 - 5.479)*** | ref |
| | | 15-24 | 30 | 92 | 206.7 | 3.067 (2.031 - 4.630)*** | 2.999 (1.945 - 0.626)*** |
| | | 25-64 | 247 | 688 | 178.5 | 2.785 (2.409 - 3.221)*** | 2.715 (2.239 - 3.291)*** |
| | | > 64 | 14 | 18 | 28.6 | 1.286 (0.639 - 2.585) | 1.243 (0.602 - 2.563) |
| | Functional | Working | 286 | 779 | 172.4 | 2.724 (2.379 - 3.119)*** | ref |

| | | | | | | | |
|---|---|---|---|---|---|---|---|
| | status | Ambulatory | 14 | 58 | 314.3 | 4.143 (2.311 - 7.426)*** | 3.186 (1.707 - 5.947) |
| | | Bedridden | 0 | 1 | - | - | - |
| | Development milestone | Appropriate | 7 | 13 | 85.7 | 1.857 (0.741 - 4.655) | ref |
| | | Delay | 1 | 2 | 100.0 | 2 (0.181 - 22.056) | 1.128 (0.076 - 16.837) |
| | | Regression | 0 | 0 | | - | - |
| | WHO stage | One | 223 | 631 | 183.0 | 2.83 (2.429 - 3.296)*** | ref |
| | | Two | 49 | 126 | 157.1 | 2.571 (1.849 - 3.577)*** | 0.906 (0.627 - 1.311) |
| | | Three | 33 | 88 | 166.7 | 2.667 (1.787 - 3.979)*** | 0.926 (0.586 - 1.463) |
| | | Four | 3 | 7 | 133.3 | 2.333 (0.603 - 9.023) | 0.565 (0.135 - 2.362) |
| **Number of people with good ART** | Sex | Female | 22678 | 12314 | -45.7 | 0.543 (0.531 - 0.555)*** | ref |
| | | Male | 12718 | 6845 | -46.2 | 0.538 (0.523 - 0.554)*** | 0.967 (0.932 - 1.004) |
| | Age | < 15 | 2692 | 1560 | -42.1 | 0.579 (0.544 - 0.617)*** | ref |
| | | 15-24 | 2458 | 1404 | -42.9 | 0.571 (0.535 - 0.609)*** | 0.592 (0.553 - 0.634)*** |

| | | | | | | | |
|---|---|---|---|---|---|---|---|
| **adherence** | | 25-64 | 29585 | 15869 | -46.4 | 0.536 (0.526 - 0.547)*** | 0.554 (0.541 - 0.568)*** |
| | | > 64 | 661 | 326 | -50.7 | 0.493 (0.432 - 0.563)*** | 0.512 (0.447 - 0.586)*** |
| | Functional status | Working | 31546 | 17071 | -45.9 | 0.541 (0.531 - 0.551)*** | ref |
| | | Ambulatory | 3380 | 1801 | -46.7 | 0.533 (0.503 - 0.564)*** | 0.665 (0.624 - 0.709)*** |
| | | Bedridden | 67 | 27 | -59.7 | 0.403 (0.258 - 0.629)*** | 0.804 (0.499 - 1.297) |
| | Development milestone | Appropriate | 302 | 189 | -37.4 | 0.626 (0.522 - 0.751)*** | ref |
| | | Delay | 71 | 53 | -25.4 | 0.746 (0.523 -1.065) | 1.103 (0.730 - 1.667) |
| | | Regression | 30 | 18 | -40.0 | 0.6 (0.334 - 1.076) | 0.857 (0.461 - 1.595) |
| | WHO stage | One | 25364 | 13876 | -45.3 | 0.519 (0.508 - 0.530)*** | ref |
| | | Two | 5293 | 2699 | -49.0 | 0.624 (0.597 - 0.651)*** | 0.928 (0.882 - 0.977)** |
| | | Three | 4321 | 2389 | -44.7 | 0.576 (0.549 - 0.605)*** | 1.045 (0.987 - 1.106) |
| | | Four | 418 | 195 | -53.3 | 0.456 (0.382 - 0.545)*** | 0.900 (0.747 - 1.086) |

IRR, Incidence Risk Ratio; OR, Odds ratio; *$P$<0.05; **$P$<0.01; ***$P$<0.001; ref, reference; Negative sign (-) indicate percentage reduction

**Table S4.** HIV Disease Progression during COVID-19 Pandemic based on Socio-demographic Characteristics and Clinical Conditions of PLHIV

| Indicators | Baseline Variables | | Before COVID-19 (Jan 2018 to Dec 2019) | During COVID-19 (Jan 2020 to Dec 2021) | Change in percentage (%) | IRR (95%CI), Comparing indicators before and during COVID-19 | OR (95%CI), Comparing subgroups with the reference group |
|---|---|---|---|---|---|---|---|
| **Percentage of Viral suppression** | Sex | Female | 87.62 | 63.09 | -28.00 | 0.425 (0.390 - 0.463)*** | ref |
| | | Male | 83.08 | 60.86 | -26.75 | 0.481 (0.431 - 0.537)*** | 1.135 (0.985 - 1.307) |
| | Age | < 15 | 69.86 | 48.84 | -30.09 | 0.432 (0.321 - 0.579)*** | ref |
| | | 15-24 | 75.90 | 51.47 | -32.19 | 0.473 (0.355 - 0.628)*** | 1.183 (0.734 - 1.906) |
| | | 25-64 | 87.70 | 64.04 | -26.98 | 0.446 (0.415 - 0.479)*** | 1.114 (0.752 - 1.649) |
| | | > 64 | 95.31 | 67.74 | -28.93 | 0.344 (0.209 - 0.565)*** | 0.833 (0.443 - 1.564) |
| | Functional status | Working | 86.96 | 62.63 | -27.98 | 0.444 (0.413 - 0.476)*** | ref |
| | | Ambulatory | 77.64 | 61.54 | -20.74 | 0.478 (0.371 - 0.617)*** | 1.044 (0.742 - 1.469) |
| | | Bedridden | 91.67 | 66.67 | -27.27 | 0.182 (0.040 - 0.820)* | 0.530 (0.103 - 2.738) |

| | | | | | | | |
|---|---|---|---|---|---|---|---|
| | Development milestone | Appropriate | 52.38 | 28.57 | -45.46 | 0.364 (0.116 - 1.142) | ref |
| | | Delay | 50 | 20 | -60.00 | 0.333 (0.035 - 3.204) | 0.576 (0.037 - 8.998) |
| | | Regression | - | - | - | - | - |
| | WHO stage | One | 87.37 | 62.57 | -28.39 | 0.433 (0.399 - 0.469)*** | ref |
| | | Two | 83.68 | 60.86 | -27.27 | 0.463 (0.388 - 0.551)*** | 1.066 (0.878 - 1.295) |
| | | Three | 80.60 | 62.36 | -22.63 | 0.513 (0.424 - 0.617)*** | 1.184 (0.954 - 1.470) |
| | | Four | 86.67 | 57.14 | -34.07 | 0.308 (0.161 - 0.588)*** | 0.793 (0.387 - 1.628) |
| **HIV-related Death** | Sex | Female | 24 | 81 | 237.5 | 3.375 ( 2.140 - 5.322)*** | ref |
| | | Male | 36 | 61 | 69.4 | 1.694 (1.122 - 2.558)* | 0.622 (0.325 - 1.192) |
| | Age | < 15 | 5 | 6 | 20.0 | 1.2 (0.366 - 3.932) | ref |
| | | 15-24 | 3 | 12 | 300.0 | 4 (1.129 - 14.175)* | 14.067 (2.991 - 66.156)** |
| | | 25-64 | 49 | 118 | 140.8 | 2.408 (1.726 - 3.360)*** | 8.532 (3.666 - 19.856)*** |
| | | > 64 | 3 | 6 | 100.0 | 2 (0.500 - 7.997) | 6.694 (1.284 - 34.903)* |

| | | | | | | | |
|---|---|---|---|---|---|---|---|
| | Functional status | Working | 18 | 79 | 338.9 | 4.389 (2.631 - 7.323)*** | ref |
| | | Ambulatory | 20 | 39 | 95.0 | 1.95 (1.137 - 3.343)* | 1.096 (0.452 - 2.656) |
| | | Bedridden | 19 | 21 | 10.5 | 1.105 (0.594 - 2.056) | 0.806 (0.207 - 3.147) |
| | WHO stage | One | 8 | 55 | 587.5 | 6.875 (3.275 - 14.433)*** | ref |
| | | Two | 11 | 28 | 154.5 | 2.545 (1.267 - 5.113)** | 0.361 (0.120 - 1.083) |
| | | Three | 16 | 32 | 100.0 | 2 (1.097 - 3.645)* | 0.303 (0.092 - 0.999)* |
| | | Four | 25 | 27 | 8.0 | 1.08 (0.627 - 1.861) | 0.194 (0.044 - 0.851)* |

IRR, Incidence Risk Ratio; OR, Odds ratio; **P*<0.05; ***P*<0.01; ****P*<0.001; ref, reference; Negative sign (-) indicate percentage reduction
